# Effect of Four Bleaching Regimens on Color Changes and Microhardness of Dental Nanofilled Composite

**DOI:** 10.1155/2009/313845

**Published:** 2009-11-12

**Authors:** Simone Xavier Silva Costa, Anne Buss Becker, Alessandra Nara de Souza Rastelli, Leonor de Castro Monteiro Loffredo, Marcelo Ferrarezi de Andrade, Vanderlei Salvador Bagnato

**Affiliations:** ^1^Department of Restorative Dentistry, Araraquara School of Dentistry, University of São Paulo State, 1680 Humaitá Street 14801-903, Araraquara, SP, Brazil; ^2^University Center Franciscano (UNIFRA), 1250 Andradas Street 97010-030, Santa Maria, RS, Brazil; ^3^Physics Institute of São Carlos, University of São Paulo, 400 Trabalhador São Carlense Avenue 13560-970, São Carlos, SP, Brazil

## Abstract

*Objective*. The purpose of this study was to compare the color changes and microhardness of a nanocomposite after four bleaching regimens. *Materials*. Twenty-five specimens (*n* = 25) were made with a nanocomposite resin (Filtek Supreme XT). The specimens were divided into five groups equally (*n* = 5): bleaching groups and control group, as follows: G1: artificial saliva at 37°C; (control) G2: hydrogen peroxide (HP) at 7%; G3: hydrogen peroxide (HP) at 35%; G4: carbamide peroxide (CP) at 10%; G5: carbamide peroxide (CP) 35%. Color measurements were made with spectrophotometer using CIELAB color scale. The Vickers hardness (VHN) measurements were performed at the top surface. The data were analyzed with two-way Analysis of Variance. *Results*. ΔE and VHN mean values into the groups were not statistically different, however, the VHN mean values before and after storage and bleaching showed statistically significant differences. *Conclusion*. Nanocomposite samples showed no significant alteration (color and microhardness) after bleaching. Thus, no replacement of restorations is required after bleaching.

## 1. Introduction

The use of bleaching agents to improve the appearance of natural dentition has become a popular procedure since their introduction by Haywood and Heymann [1]. Currently, the bleaching agents are based primarily on hydrogen peroxide (HP) or its compounds such as carbamide peroxide (CP). The bleaching agents provide whitening of tooth structure through decomposition of peroxides into unstable free radicals. These radicals further breakdown into large pigmented molecules either through an oxidation or a reduction reaction. The oxidation/reduction process changes the chemical structure of interacting organic substances of tooth, which results in color change [2, 3]. However, the application of bleaching agents can affect human teeth and restorative materials. 

Effect of bleaching on dental restorative materials in general has been reviewed recently [4]. Due to their organic matrix, composite resin materials especially are more prone to chemical alteration compared to inert metal or ceramic restorations [5]. Although the application of low concentrations of CP on tooth structure causes minimal surface changes [6, 7], however high-concentration solutions modify the enamel surface [8]. Bleaching agents also deteriorate the surface of existing composite restorations and induce bacterial adhesion [9]. 

The use of bleaching agents is widespread, however studies investigating the effect of bleaching treatments on the microhardness of restorative materials have reported conflicting results [10–14].

The effects of CP (home bleaching) on the surface microhardness are material-dependent [13, 15–17]. Significant increase in the surface microhardness of glass-ionomer cement after exposure to 15% CP was verified [17, 18]. However, bleaching agents produced a significant microhardness decrease for compomer [11, 12]. No significant surface microhardness changes were found after application of 15% CP for composite resin [17]. However, other studies show that the bleaching agents do not reduce the microhardness of the restorative materials [19, 20].

Bleaching agents may affect the color of existing composite restorations. The color change of polyacid-modified composites is generally higher than the change recorded for hybrid and macrofilled composites. Thus, the effect of bleaching agents on the color of restorative materials are material-dependent [21]. 

Several studies show the effects of peroxide-based bleaching agents on dental restorative materials. Generally, these studies indicate that the effects of bleaching agents are minor and clinically insignificant as slight roughening of composite resin surfaces. The bleaching agents do not perceptibly change the shade of tooth-colored restorative materials [22].

Recently, a nanofilled resin composite with primary filler size of 5–20 nm was introduced [23]. A composite dental filling material that could be used in all areas of the mouth with high initial polish and superior polish retention (typical of microfills), as well as excellent mechanical properties suitable for high stress–bearing restorations (typical of hybrid composites) [24]. Because of these properties the nanofilled composite may present smaller changes of bleaching agents. Its surface properties may be different from those of hybrid composites. 

Although there are several reports on the effect of home bleaching systems on composites [25, 26], little is known about the effects of the in-office bleaching technique on the restorative materials [4, 11]. There is limited data in literature on the effects of bleaching systems on microhybrid and nanofilled composite resins. Thus, the purpose of this study was to evaluate the effects of four bleaching regimens on color changes and microhardness of nanofilled composite resins.

## 2. Material and Methods

The light-cured composite material used in the study was Filtek Supreme XT (batch number 6BN ET-2181/01, 3M ESPE, Dental Products St. Paul, Minn, USA) (Table 1). 

For the color and microhardness measurements specimens with 8 mm in diameter and 1 mm thickness were made in a metallic mold. The composite resin was packed into the mold, with the upper and lower surfaces covered with acetate matrix strips. The specimens were light-cured during 40 seconds by a light-curing unit- (LCU) based on LED (LEC 1000, MM Optics, São Carlos, SP, Brazil). The LCU was calibrated before light-curing process of the composite resin, and the power density of 500 mW/cm^2^ was obtained by the use of a power meter (Fieldmaster, Coherent-model n° FM, set n° WX65, part n° 33-0506, USA).

Following light-curing, the specimens were removed from the molds and placed at 37°C distilled water for 24 hours to assure complete polymerization.

### 2.1. Color Measurements

Initial quantitative color (ΔE) measurements were performed by the use of a spectrophotometer (Pocket Spee-ColorQA Pro, PocketSpee Techonologies Inc., Denver, Colo, USA). During baseline measurements, three measurements were performed for each specimen, and the mean of the readings was calculated. The mean of each specimen was calculated by use of the CIE Lab uniform color scale.

The magnitude of the total color difference is represented by a single number ΔE^(CIE,1971)^ : ΔE = [(ΔL*)^2^ + (Δa*)^2^ + (Δb*)^2^]^1/2^, where L* represents lightness, a* redness-greenness and b* yellowness-blueness. This formula provides numeric data that represent the differences in color perceived between 2 objects.

The specimens were then randomly divided into 5 groups (*n* = 5):

- Group 1: stored in artificial saliva at 37°C for three weeks and served as control;
- Group 2: specimens were treated with hydrogen peroxide at 7% for four hours per day for two weeks;
- Group 3: specimens were treated with hydrogen peroxide at 35% (three sessions of 30 min each at intervals of one week);
- Group 4: specimens were treated with carbamide peroxide at 10% for four hours per day for two weeks;
- Group 5: specimens were treated with carbamide peroxide at 35% (three sessions of 30 minutes each at intervals of one week).

During the test period, the specimens were kept at 37°C and 100% relative humidity. Each day after the active treatment period the specimens were rinsed with distilled water to remove the bleaching agents and stored in artificial saliva. At the end of the bleaching regimens, color changes measurements of the control and the test groups were obtained as previously described. All specimens were measured three times and the average values were calculated.

### 2.2. Microhardness Measurements

After curing and storage for 24 hours the top surfaces of all specimens were then polished with a sequential series of 3 (medium, fine, and superfine) Sof-Lex disks (3M ESPE) and a slow-speed handpiece. The specimens were then randomly divided into 5 groups (*n* = 5) as already reported for the color's assessment.

The specimens were blotted dry and positioned beneath the indenter of a microhardness tester Micromet 2100 (Buehler, Lake Bluff, Ill, USA). A 50 g load was applied through the indenter, with a dwell time of 30 seconds. This method depended on visualization of the surface indentations through the microscope of the testing machine. The length of the diagonal of each indentation was measured directly from the graduated eyepiece of the microscope in the testing machine. Four indentations were made at random on the top surface of each specimen, and a mean value was calculated as the microhardness for that specimen. Microhardness was measured at 24 hours after polymerization (baseline), at the end of the bleaching regimens (three weeks) on the same specimen.

### 2.3. Statistical Analysis

The data were analyzed by Analysis of Variance (ANOVA) with Stata Software (Stata Statistical Software: Release 8.0, College Station, Stata Corporation, Tex, USA) at level significance of 5%.

## 3. Results

### 3.1. Color Measurements

Table 2 shows the mean ΔE values for the different groups. The results showed that the differences were not statistically significant among groups (*P* = .4989). The ΔE values of each bleaching regimens were analyzed by Analysis of Variance and the data showed that all four bleaching regimens had no significant effect on the color of nanofilled composite resin Filtek Supreme XT (3M ESPE, Dental Products St. Paul, Minn, USA).

**Figure 1:**
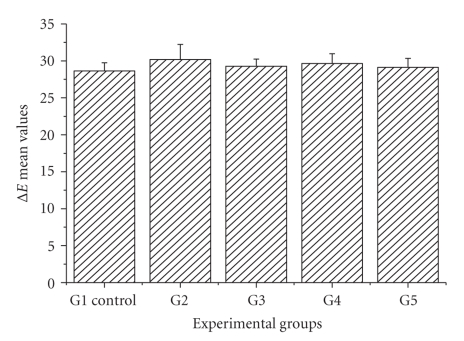
Mean ΔE values for all Groups (G1: control; G2: Hydrogen peroxide 7%; G3: Hydrogen peroxide 35%; G4: Carbamide peroxide 10%; G5: Carbamide peroxide 35%).

Comparing the mean color changes performed in each one of the five groups, it can be seen that they were homogeneous and this finding can point out to the same results in the use of these four bleaching regimens (Figure 1).

### 3.2. Microhardness Measurements

Means and standard deviations of the VH of the specimens at baseline and after bleaching regimens are shown in Tables 3 and 4. The difference between the VH values (baseline and after bleaching) were statistically significant (*P* < .01). The VH values after storage and bleaching was significantly lower in comparison to baseline values.

All solutions, either storage (artificial saliva) or treatment (bleaching agents) affected VHN of the restorative material at all the levels evaluated. However, storage in artificial saliva and bleaching regimens promoted significant decrease in VHN mean values for all Groups tested.

**Figure 2:**
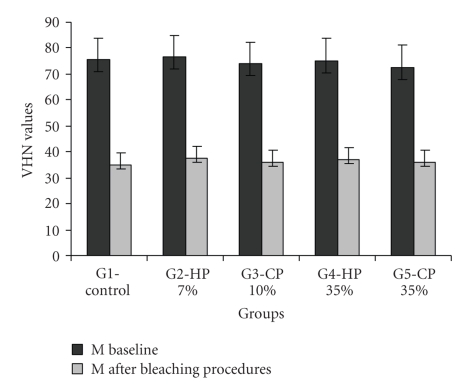
Microhardness mean values (M) and standard deviation (SD) for all Groups baseline and after bleaching procedures (G1: control; G2: Hydrogen peroxide 7%; G3: Hydrogen peroxide 35%; G4: Carbamide peroxide 10%; G5: Carbamide peroxide 35%).

Similar VHN values was observed in bleached samples, that is, from specimens treated with HP (7% and 35%) and CP (10% and 35%), as well as samples of the positive control. Thus, after bleaching regimens, no significant differences were found among Groups. The Figure 2 shows the results of microhardness for baseline and after bleaching procedures.

## 4. Discussion

Today “whiter teeth” is the most common aesthetic request from dental patients and tooth whitening is a relatively noninvasive approach to achieving this goal. As bleaching of teeth has become extremely popular, the effect of bleaching on aesthetic appearance of dental materials must be considered. This complicates the process of trying to establish and maintain good color match between the dental restoration and the adjacent tooth structure. Changes in the chemical and morphological structure of restorations must be of concern when bleaching is used as a whitening treatment [8, 26, 27].

The contemporary tooth bleaching technique is based primarily on the oxidation by hydrogen peroxide or one of its precursors, and those are often used in combination with an activating agent such as heat or light. The commercial products of tooth bleaching are usually fabricated in a gel form and can be administered professionally in dental clinics (in-office bleaching) or used by patients with trays at home (home bleaching). Current available agents are usually based on 6–20 and 25%–40% peroxide gels for home and in-office whitening, respectively [8, 27].

Patients seeking bleaching treatment may have teeth restored with different kinds of aesthetic restorative material. It is possible that chemical softening, resulting from bleaching, may affect the clinical durability of these materials. Drastic color changes to existing restorations may compromise esthetics; therefore it is important to understand the effect of bleaching agents on the color of restorative materials. The interaction between the bleaching agent and restorative material is of clinical significance because the color change may be noticed by the patient [17, 21].

After the application of bleaching agents, whitening of tooth results from oxidation of organic substances by free radicals [3]. In case of dental composite resins, bleaching agents may have an influence on resin matrix, filler, or both. However, fillers are basically glass or ceramic, and therefore the influence of hydrogen peroxide on fillers would be very small. Instead, the resin matrix may be chemically degraded by the concentrated or repeated application of hydrogen peroxide [28]. 

Several studies have evaluated the effects of bleaching on dental hard tissues [7, 29–32]. A recently published review showed that tooth-bleaching agents may have negative effects on dental restorative materials. The effects included changes on surface morphology and also in their chemical and physical properties [4]. Yalcin and Gurgan [26], showed that the gloss of tooth colored restorative materials were significantly decreased by the bleaching regimens. Thus, bleaching agents should not be used indiscriminately when these restorations are present. 

Nevertheless, because literature presents controversial findings, the influence on physical properties and surface morphology of dental materials needs a closer approach. Some authors have reported microstructural changes and decreased hardness in restorative materials after bleaching [13, 25], while other studies found only slight changes or no changes [13, 33, 34]. Furthermore, the interaction between office solutions and both, teeth and restorations still raises concern, once higher peroxide concentrations could worsen possible harmful effects [35]. 

This statement is in accordance with Monaghan et al. [36, 37] where highly concentrated office bleaching systems affected the color of composite resin, however low concentrations of home bleaching systems did not. 

Cooley and Burger [38] evaluated composite resins for changes in surface hardness, roughness, and lightness after exposure to 10% carbamide peroxide gels and found that these three parameters increased significantly after exposure. Cullen et al. [39] reported that 10% carbamide peroxide and 30% hydrogen peroxide had no significant effect on tensile strength of highly filled composite resins. However, microfilled composite resins were significantly affected by 30% hydrogen peroxide, resulting in a reduction in tensile strength. Canay and Cehreli [21] showed that 10% hydrogen peroxide provided more color changes of composite resins compared with 10% carbamide peroxide, and the color change of all composite resins bleached with hydrogen peroxide solution was clinically detectable to the naked eye. Lima et al. [40] showed that 16% carbamide peroxide reduced the microhardness of the hybrid composite surface, independent of the type of light source used.

The bleaching regimens used in this study did not have any significant effect on the color changes and microhardness for the material tested. These findings coincide with the results of Kim et al. [28] where the influence of tooth-whitening film and strip on the color and surface roughness of dental composite resins was negligible. Also, there was no difference in the color change and surface roughness according to the type of composite resin, whether nanofilled or microhybrid. In other study [17], the 15% carbamide peroxide did not cause any significant effect on the surface microhardness for all composite resins tested (Filtek Z-350 and Filtek P-60/3M ESPE) and the effects of 15% carbamide peroxide on the surface microhardness were material-dependent.

The nanofilled composite was development for used in all areas of the mouth with high initial polish and superior polish retention (typical of microfills), as well as excellent mechanical properties suitable for high stress–bearing restorations (typical of hybrid composites). This recent tooth-colored restorative material was used in this study. Changes in the structure or composition of this restorative material may have provided more resistant surfaces against bleaching treatments [19]. The composite resin Filtek Supreme XT (3M ESPE) as a nanofilled composite has an average particle size 0.6–1.4 microns. This may be another reason why Filtek Supreme XT (3M ESPE) with smaller filler size has the highest polishing and consequently, smaller effect from bleaching agents. 

The results after bleaching regimens showed that bleaching did not affect the color and microhardness of composite resin, even after 4 hours of application of hydrogen peroxide 7% and carbamide peroxide 10% over a period of 14 days (home bleaching) and even after 30 minutes application of hydrogen peroxide and carbamide peroxide 35% over a period for three sessions (office bleaching). These results are consistent with previous studies for the low concentration of carbamide peroxide bleaching products [21, 28], however, not for the high concentrated bleaching agent. 

There is controversy about the impact of low concentrated 10%–16% carbamide peroxide gels on surface microhardness of restorative composite materials. In some investigations softening of composite resins was associated with the application of home-bleaching gels [25, 41]. Other investigations revealed no significant hardness changes [10, 42] due to application of home-bleaching gels or even an increase in surface hardness [13, 43]. In-office tooth whiteners (35% carbamide peroxide or 35% hydrogen peroxide) did not significantly affect hardness and tensile strength of composite materials [44, 45].

Such wide variations in data suggest that some tooth colored restorative materials may be more susceptible to alteration and some bleaching agents are more likely to cause those alterations [46]. The discrepancies between these studies may be explained by the differences inexperimental methodologies, bleaching agents applied [47], and restorative materials used [13]. The frequency with which the bleaching agents were changed may also contribute to the disparity between the results of the studies. In the present study, bleaching products were applied with clinically relevant bleaching regimes according to manufacturer's instructions. Application periods selected were 4 hours per day for the home-bleaching and 30 minutes for session for the office-bleaching. This was different from several other bleaching studies in which materials were continuously exposed to bleaching products for several days to simulate cumulative effects over time [11, 12].

In vitro studies are limited in their attempt to simulate clinical conditions. It was shown that peroxide levels in bleaching products are depleted during use depending on the in vivo situation [47]. In this study, the bleaching agents were not diluted or buffered with any water content such as saliva or distilled water during bleaching treatments, as in most other studies [13, 44–46]. Only after regimens bleaching the specimens were stored in artificial saliva. Storage of composite specimens in saliva between incubation with the bleaching material was done to simulate the clinical situation. It is conceivable that storage in saliva might have modified or attenuated the whitening agent impact by the formation of a surface-protection salivary layer on the restorative material [48].

The bleaching mechanism for teeth is that the active agents (peroxide solutions) can flow freely through the enamel and dentin and oxidize the pigments in the teeth [49]. The results of the present study showed that the color change of composite resin after bleaching was probably due to superficial cleansing of the specimens, not intrinsic color change.

Although bleaching agents can successfully remove the exterior staining from composite resins, they will not bleach them, whereas they can effectively bleach teeth [6, 50]. Therefore, after bleaching, the composite resin restoration may not match the surrounding bleached tooth structure. Also, bleaching can increase the surface roughness of composite resins; therefore, the restoration may stain more easily after bleaching [13, 16]. Bleaching agents should be used cautiously to remove the exterior stain on the surface of composite resin restorations.

Colorimetry is a branch of the science of color based on the digital expression of the color perceived from the object. In assessing chromatic differences, generally 2 color systems are used: Munsell Color System and Standard Comission Internationale de L'Eclairage (CIE Lab) Color System. The American Dental Association recommends the use of CIE Lab color differential system [51]. According to this system, all colors in nature are obtained through blending of 3 basic colors, red, blue and green, in various proportions [21]. 

For standardized and reproducible evaluation of color changes of restorative materials, colorimeters are used analyzing L*a*b* values according to the CIELab-system [52, 53]. It has been claimed that under clinical conditions in the mouth, DE color differences have been reported to be relevant and perceptible only when higher than 3.3 [54] or 3.6 [55]. In this study the CIELab system was used.

Within the results of the present study, it can be summarized that replacement of the tested restorative materials is not required after in-office bleaching. Polydorou [20] said that there is no sufficient reason to indicate the replacement of restorations, except the cases that have esthetic involvement. Thus, patients should be advised that existing composite restorations may not match the natural teeth after bleaching, and replacement may be required.

## 5. Conclusions

The results obtained for this study indicate that color change and microhardness in the nanofilled composite after bleaching (home-bleaching and office-bleaching) was not perceptible or significant. Therefore, no replacement of restorations is required after bleaching.

## Figures and Tables

**Table 1:** Characteristics of restorative material used in the study (manufacturers' informations).

Material	Manufacturer	Shade	Material type	Matrix	Filler size	Filler Volume
Filtek Supreme XT	3M ESPE	A_3_E	Nanofilled composite	Bis-GMA, Bis-EMA, UDMA, TEGDMA.	Agglomerated/non-aggregated of 75 nm silica nanofiller and a loosely bound agglomerate silica nanocluster consisting of agglomeratesof primary silica nanoparticles of 75 nm size fillers. The cluster size range is 0.6–1.4 microns.	72.5%

**Table 2:** Mean and standard deviation (±sd) ΔE values for all experimental groups.

Experimental groups	Mean	Standard deviation (±sd)
1 (Control)	28.64	1.10
2 (Hydrogen peroxide 7%)	30.18	2.05
3 (Hydrogen peroxide 35%)	29.27	0.97
4 (Carbamide peroxide 10%)	29.65	1.33
5 (Carbamide peroxide 35%)	29.13	1.21

**Table 3:** Mean values and standard deviation (±sd) for VHN (baseline).

Bleaching regimens	Mean values (±sd)
G1	75.6 (±6.0)
G2	76.6 (±4.6)
G3	75.2 (±5.3)
G4	74.0 (±8.4)
G5	72.7 (±6.7)

**Table 4:** Mean values and standard deviation (±sd) for VHN (after bleaching).

Bleaching regimens	Mean values (±sd)
G1	34.9 (±1.6)
G2	37.4 (±2.6)
G3	36.1 (±3.3)
G4	37.1 (±1.4)
G5	36.1 (±2.5)
